# Inhibitory effect of gut bacteria from the Japanese honey bee, Apis cerana japonica, against Melissococcus plutonius, the causal agent of European foulbrood disease

**DOI:** 10.1093/jis/14.1.129

**Published:** 2014-09-15

**Authors:** Meihua Wu, Yuya Sugimura, Kyoko Iwata, Noriko Takaya, Daisuke Takamatsu, Masaru Kobayashi, DeMar Taylor, Kiyoshi Kimura, Mikio Yoshiyama

**Affiliations:** 1 Honey Bee Research Unit, Animal Breeding and Reproduction Research Division, National Institute of Livestock and Grassland Science, NARO, 2 Ikenodai, Tsukuba, Ibaraki 305-0901, Japan; 2 Bacterial and Parasitic Diseases Research Division, National Institute of Animal Health, NARO, 3-1-5 Kannondai, Tsukuba, Ibaraki 305-0856, Japan; 3 Graduate School of Life and Environmental Sciences, University of Tsukuba, 1-1-1 Tennodai, Tsukuba, Ibaraki 305-8572, Japan; 4 Technical Services for Diagnosis, Diagnostic Section, Center for Animal Disease Control and Prevention, National Institute of Animal Health, NARO, 3-1-5 Kannondai, Tsukuba, Ibaraki 305-0856, Japan; 5 The United Graduate School of Veterinary Sciences, Gifu University, Gifu, Gifu 501-1193, Japan

**Keywords:** microbial flora, honey bee disease, probiotic

## Abstract

European foulbrood is a contagious bacterial disease of honey bee larvae. Studies have shown that the intestinal bacteria of insects, including honey bees, act as probiotic organisms. Microbial flora from the gut of the Japanese honey bee, *Apis cerana japonica* F. (Hymenoptera: Apidae) *,* were characterized and evaluated for their potential to inhibit the growth of *Melissococcus plutonius* corrig. (ex White) Bailey and Collins (Lactobacillales: Enterococcaceae) *,* the causative agent of European foulbrood. Analysis of *16S rRNA* gene sequences from 17 bacterial strains isolated by using a culture-dependent method revealed that most isolates belonged to *Bacillus, Staphylococcus,* and *Pantoea.* The isolates were screened against the pathogenic bacterium *M. plutonius* by using an in vitro growth inhibition assay, and one isolate (Acja3) belonging to the genus *Bacillus* exhibited inhibitory activity against *M. plutonius.* In addition, in vivo feeding assays revealed that isolate Acja3 decreased the mortality of honey bee larvae infected with *M plutonius,* suggesting that this bacterial strain could potentially be used as a probiotic agent against European foulbrood.

## Introduction

European honey bees *(Apis mellifera* L. (Hymenoptera: Apidae)) play a critical role in human well-being, not only through the provision of honey but also by pollinating a great variety of crop plants. Fifty-two of the 115 most common global food commodities depend on honey bee pollination to produce fruit and seeds ( Klein et al. 2007 ). In addition, the value of agricultural products derived from plants pollinated by honey bees is estimated to be around $15 billion per year in the U.S. alone ( Morse and Calderone 2000 ). Consequently, the marked deterioration in the colony health of managed honey bee hives has become a major concern in many countries around the world in recent years (vanEngelsdorp and Meixner 2010). One of the major reasons for this decline is considered to be the combined effect of several honey bee diseases ( Genersch 2010 ).

European foulbrood is a serious disease of honey bee larvae that is caused by the Gram-positive bacterium *Melissococcus plutonius* corrig. (ex White) Bailey and Collins (Lactobacillales: Enterococcaceae) ( Bailey and Collins 1982 , Bailey 1983 , Forsgren et al. 2005 ). This infectious and contagious bacterial disease has caused extensive damage to the global apiculture industry ( Belloy et al. 2007 , Forsgren 2010 ). Current control measures for the disease consist of treatment with antibiotics, such as oxytetracycline hydrochloride, which inhibits *M. plutonius* replication ( Thompson and Brown 2001 ). However, the use of antibiotics in apiculture is a major concern, not only because humans will consume antibiotic residues in the resulting honey bee products ( Mutinelli 2003 ), but also because these antibiotics are toxic to honey bee broods ( Pettis et al. 2004 , Thompson et al. 2005 ) and beneficial honey bee microflora ( Vasquez et al. 2012 ). The emergence of oxytetracycline-resistant strains of *Paenibacillus larvae* (White) (Bacillales: Paenibacillaceae), the etiological agent of American foulbrood, has also become a serious problem ( Miyagi et al. 2000 ). In combination, these factors have contributed to a general decrease in the use of antibiotics in apiculture and even a ban in the European Union ( de Graaf et al. 2006 ). There is thus an urgent need to develop novel and alternative disease management techniques for controlling European foulbrood.

As in other animals, the gastrointestinal tract of honey bees is a complex ecosystem that harbors a diverse array of microbial communities ( Gilliam 1997 , Martinson et al. 2011 ). Studies have demonstrated that bacterial probiotics can induce an immune response ( Evans and Lopez 2004 ) and contribute toward maintaining a healthy bee colony ( Pătruică and Mot 2012 ). Consequently, gastrointestinal bacteria have received considerable interest for their potential application as alternative disease control agents in apiculture. Several attempts to isolate bacteria from honey and honey bee guts have been undertaken in the search for probiotic agents against *P. larvae* ( Alippi and Reynaldi 2006 , Audisio et al. 2011 ) and *Ascosphaera apis* (Maasen ex Claussen) L.S. Olive & Spiltoir (Ascosphaerales: Ascosphaeraceae), which causes chalkbrood disease ( Reynaldi et al. 2004 ).

A recent study reported that lactic acid bacteria isolated from honey bees exhibited antagonistic effects toward *M. plutonius* ( Vasquez et al. 2012 ). However, European foulbrood has not yet been systematically studied, and the pathogenic mechanisms of the disease are currently poorly understood, primarily due to difficulties associated with artificially culturing *M. plutonius* and conducting European foulbrood experiments under laboratory conditions ( McKee et al. 2004 ). However, several of the *M. plutonius* strains that have recently been isolated and artificially cultured have induced high mortalities and European foulbrood symptoms in honey bee larvae raised in vitro ( Arai et al. 2012 ). These findings have enabled us to examine the control of European foulbrood more closely and to assess the efficacy of using intestinal probiotics in novel disease management strategies.

The Japanese honey bee, *Apis cerana japonica* F. (Hymenoptera: Apidae), a subspecies of the Asian honey bee, is native to Japan. Bacteria isolated from the gut of a Japanese honey bee were shown to inhibit *P. larvae* growth ( Yoshiyama and Kimura 2009 ). Compared with *A. mellifera* , *A. cerana* is considered to be resistant to a variety of pathogens, including American foulbrood and *Varroa* mites ( Peng et al. 1987 , Chen et al. 2000 ). As a result, this honey bee species is considered to be well suited for use as a potential source of probiotic gut bacteria that confer a tolerance to a variety of honey bee pathogens. The objective of this study was therefore to isolate bacteria from the intestinal tract of the Japanese honey bee, *A. c. japonica* , and to evaluate their antagonistic effects on *M. plutonius* . The results revealed that one of the isolates, Acja3, exhibited anti- *M. plutonius* activity in *A. mellifera* .

## Materials and Methods

### Collection of *A. c. japonica* and Isolation of Gut Bacteria

Colonies of *A. c. japonica* were reared in wooden hives in an apiary at the Honey Bee Research Unit at the National Institute of Livestock and Grassland Science in Tsukuba, Japan. Foragers of *A. c. japonica* were collected from three different colonies in July 2011. Ten returning workers from each colony were caught at hive entrances by using forceps and transferred to 1.5 ml tubes. The external surface of the forager bees was sterilized with 70% ethanol and washed with distilled water. The digestive tracts were then dissected asep-tically before being homogenized in tubes containing Brain Heart Infusion (BHI) liquid medium (Difco, Detroit, MI). Gut homogenates were then plated on BHI agar plates and incubated aerobically at 35°C for 48 h. Bacterial colonies were selected according to size, color and morphological appearance.

### DNA Sequencing of *16S rRNA* Genes

The *16S rRNA* genes of all isolates were amplified by PCR with 27F (5ʹ-AGA GTT TGA TCC TGG CTC AG-3ʹ) and 1406R (5ʹ-ACG GGC GGT GTG TAC-3ʹ) primers ( Weisburg et al. 1991 ). Polymerase chain reactions (50 µL) contained 1 U of KOD-Plus *-* DNA polymerase (Toyobo, Osaka, Japan) with 1× PCR buffer (1.5 mM MgCl _2_ ), 0.2 mM of dNTPs, and 0.5 µM of each primer. The PCR cycle consisted of an initial denaturation at 95°C for 2 min, followed by 35 cycles of denaturation at 95°C for 1 min, annealing at 60°C for 1 min, and extension at 68°C for 1 min. After separating the fragments on a 1.2% agarose gel, PCR products were extracted using a Qi-aex II Gel Extraction Kit (Qiagen, Germantown, MD) before cloning into a pGEM-T Easy Vector (Promega, Madison, WI). Ligation mixture was then transformed into One Shot ^®^ TOP10 *E. coli* (Invitrogen, Carlsbad, CA) according to the manufacturer’s instructions. Plasmid DNA was purified using a QIAprep Miniprep Kit (Qiagen). Sequencing was performed using a BigDye Terminator Cycle Sequencing Kit (Applied Biosystems, Carlsbad, CA) in combination with an automated sequencing system (Model ABI 3730, Applied Biosystems).

### Sequence and Phylogenetic Analyses

Similarities between the *16S rRNA* genes obtained in this study and published sequences were determined using the BLAST program at the National Centre for Biotechnology Information (NCBI) database ( www.ncbi.nlm.nih.gov/BLAST ). Phylogenetic relationships were estimated by performing nucleotide sequence alignments with CLUSTALX ( www.clustal.org ) and analysis with the neighbor joining (NJ) algorithm implemented in NJplot ( http://doua.prabi.fr/software/njplot ) (bootstrap value: 1,000).

### Biochemical Analysis

All bacterial isolates were characterized using an API 50CH Biochemical Kit (BioMerieux, Marcy I’Eloile, France) in combination with API 50CHB/E medium. Pure bacterial colonies from each plate were suspended in 100 µL sterile distilled water and mixed with API 50CHB/E medium. The medium inoculated with each suspension was then incubated in API 50CH strips at 35°C. Changes in color were recorded after 24 or 48 h according to the manufacturer’s instructions.

### In Vitro Inhibition Assays for *M. plutonius*

Inhibitory activities of each isolate were assayed against the *M. plutonius* DAT561 strain *,* which exhibited virulence in larvae reared in vitro ( Arai et al. 2012 ), by using a modification of a previously described diffusion technique ( Alippi and Reynaldi 2006 ). The *M. plutonius* DAT561 strain was cultured in KSBHI medium (BHI medium plus 0.15 M KH _2_ PO _4_ and 1% soluble starch) for 5 d at 35°C under anaerobic conditions in an AnearoPack System (Mitsubishi Gas Chemical Co. Inc., Tokyo, Japan). Bacterial lawns of *M. plutonius* (500 µL) were prepared on the KSBHI agar plates. Each gut bacterial isolate that had been cultured on BHI agar plates was then suspended in BHI liquid medium, and the OD _600_ was adjusted to 1.0. A sheet of filter paper (7 mm diameter) was then impregnated with each suspension (10 µL) of gut bacteria and placed on the plate with the *M. plutonius* lawn. The plates were then incubated at 35°C under anaerobic conditions for 3 d. The diameters of the inhibition zones were then measured, and the mean ± SD was calculated based on three independent experiments. BHI liquid medium was used as a negative control, and tetracycline (10 µg/mL) was used as a positive control.

### In Vivo Infectious Feeding Assays with *A. mellifera* Larvae

Larvae were collected from *A. mellifera* colonies maintained by the Honey Bee Research Unit in Tsukuba, Japan. The protocol employed for in vitro larval rearing followed the methods of Aupinel et al. (2005) with some modifications. A queen was confined in an excluder cage for 1 d, and larvae (< 24 h old) were collected from the colony on the fourth day. First-instar larvae were grafted and transferred to an artificial diet in a 24-well cell culture plate. The culture plates were kept in a plastic box and incubated at 35°C and a relative humidity of 90%. The artificial larval diet consisted of royal jelly (50%), water (37%), D-glucose (6%), D-fructose (6%), and yeast extract (1%). Larvae of experimental groups were pre-fed 50% sucrose water mixed with the same volume of a bacterial suspension of Acja3 (ca. 1 × 10 ^7^ cfu/mL), or a cell-free supernatant of Acja3 prepared from 48-h-incubated culture medium. Larvae of control groups were fed 50% sucrose water only. After 6 h, larvae in all of the groups, except the control group, were infected with *M. plutonius* DAT561 strain (ca. 1 × 10 ^7^ cfu/mL) through their normal artificial diet. Twenty-four hours after infection with *M. plutonius* , normal artificial diet was supplied for the remainder of the experiment. Larval mortality was then assessed every day under a stereomicroscope over a 5 d period. Dead larvae were distinguished by the absence of respiration, decreased body elasticity, and a change in body color from white to milky yellow. The larval survival rates were analyzed by log-rank tests to determine statistical significance.

## Results

### *16S rRNA* Gene Sequences and Phylogenetic Analysis of Isolates

**Figure 1.**
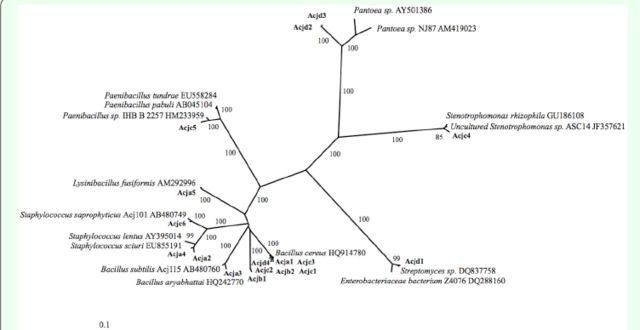
Phylogenetic tree based on *16S rRNA* gene sequences of bacteria isolated from the gut of the Japanese honey bee, *A. c. japonica.* Isolates from Yoshiyama and Kimura (2009) are Acj1 15 and Acj101. Names of the isolates obtained in this study are shown in bold. Bootstrap values > 80% (expressed as percentages of 1,000 replicates) are given at the tree nodes. Scale bar represent 0.1 nucleotide substitutions per site.

In total, 128 bacterial colonies were isolated from the gut of *A. c. japonica* by using a culture-dependent method. Of these isolates, 17 were selected based on colony size, color, and morphology, and their *16S rRNA* genes were sequenced. The 17 *16S rRNA* gene sequences were designated Acja1∽Acjd4 (GenBank accession numbers AB668062∽AB668078) and subjected to an NCBI BLAST-N search to identify sequences with the highest sequence similarities ( Table 1 ). A phylogenetic tree was constructed by NJ analysis of the 17 DNA sequences obtained in this study ( Table 1 ) as well as related sequences obtained using the BLAST-N search ( Fig. 1 ).

**Table 1:**
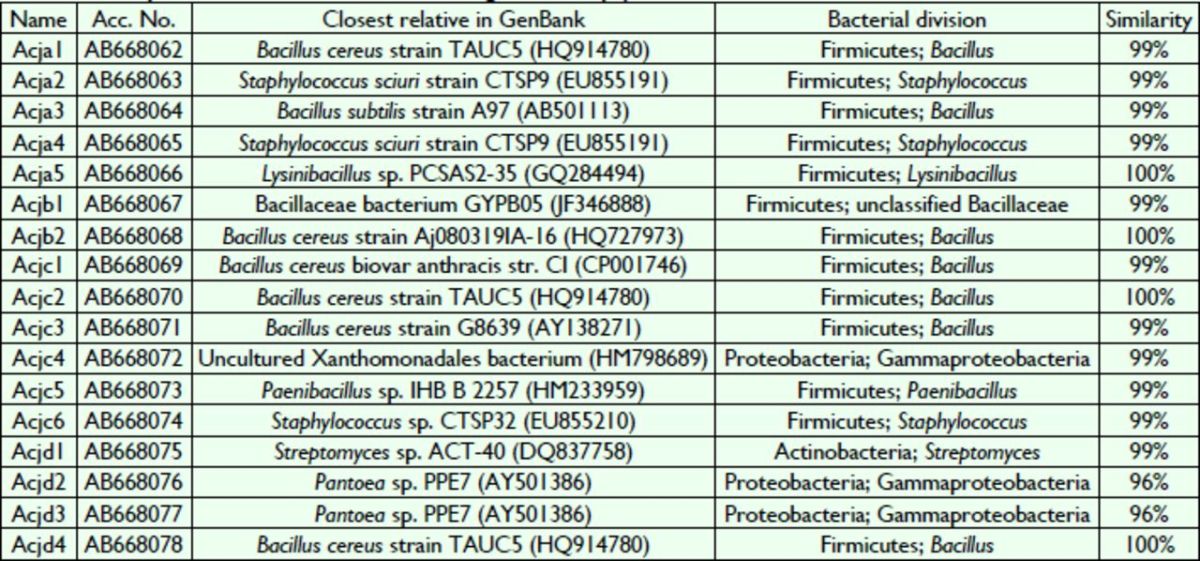
Diversity of bacterial isolates from the gut of *A. c. japonica*

One of the isolates (Acjd1) belonged to the phylum Actinobacteria, three isolates (Acjc4, Acjd2, and Acjd3) belonged to the class Gammaproteobacteria in the phylum Proteobacteria, and the remaining 13 isolates be-belonged to the phylum Firmicutes.

Isolates were then further classified into the following seven genera based on *16S rRNA* gene sequence similarities: *Bacillus, Staphylococcus, Lysinibacillus, Xanthomonas, Pae-Paenibacillus, Streptomyces,* and *Pantoea* . The results showed that eight strains from this study (Acja1, Acja3, Acjb1, Acjb2, Acjc1, Acjc2, Acjc3, and Acjd4) grouped with the main cluster, which consisted of members of the genus *Bacillus* . The Acjd2 and Acjd3 isolates were most closely related to the *Pantoea* sp. PPE7, and this association was supported by high bootstrap values. The Acjc4 isolate was closely related to unclustered Xan-thomonadales bacteria, and the Acja5 isolate formed a distinct cluster with *Lysinibacillus fusiformis* . The Acjc5 isolate was closely associated with a *Paenibacillus* sp. (HM233959), which was isolated previously from the roots of a tree.

### Carbon Utilization by the Isolated Bacteria

Carbon sources utilized by the 17 isolates were examined using an API 50CH Biochemical Kit. Although all isolates, except Acja4 and Acja5, utilized glucose and fructose ( Table 2 ), which are major components of honey, each isolate had a unique carbon source utilization profile. Although isolates Acjc2 and Acjd4 had identical *16S rRNA* gene sequences, the isolates could be distinguished from each other based on differences in their utilization of four different sugar substrates (grey backgrounds in Table 2 ), suggesting that biochemical profiles could be used to distinguish between isolates with highly similar *16S rRNA* gene sequences.

**Table 2:**
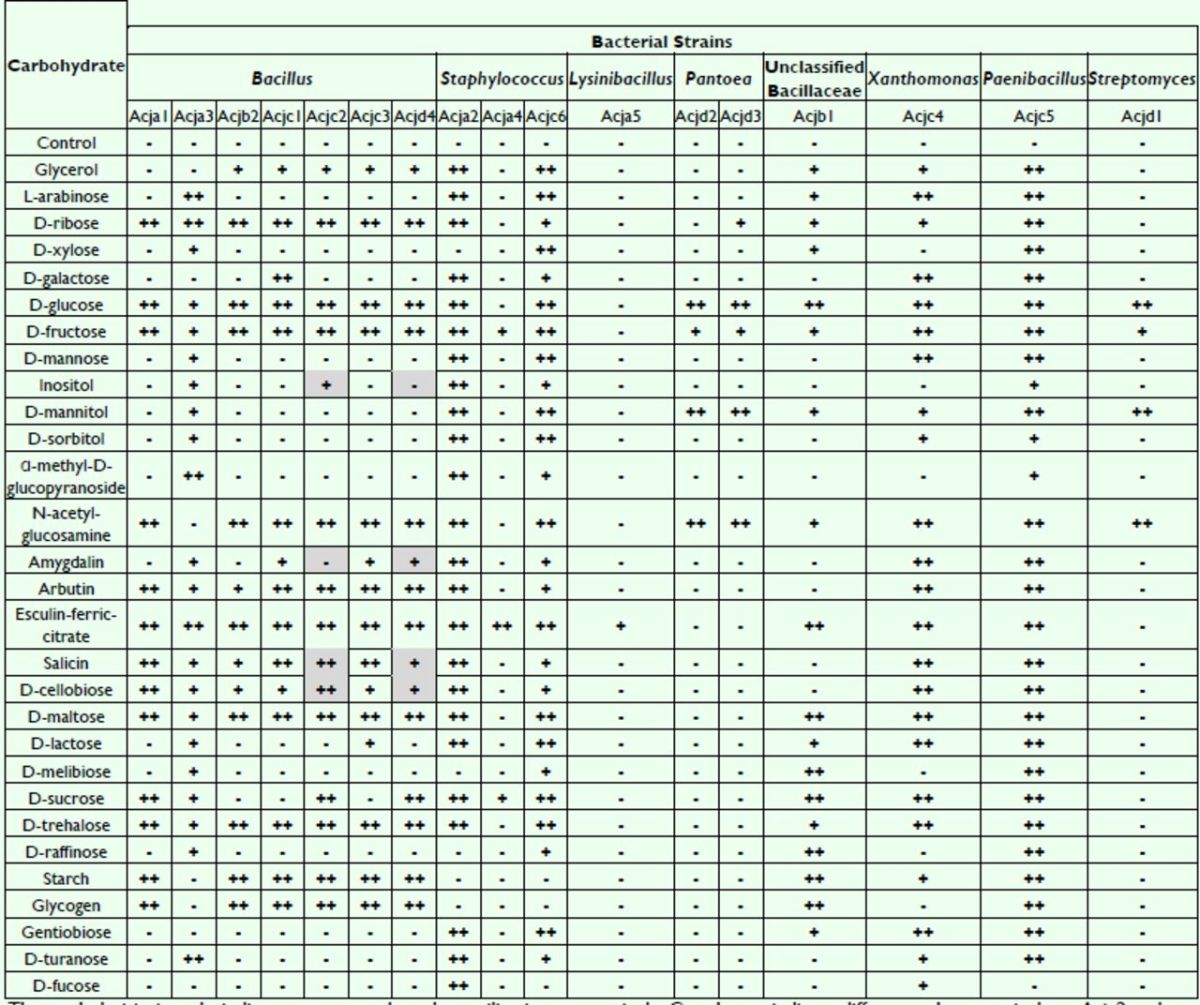
Carbon source assimilation profiles using API 50CH

The symbols ++, +, and - indicate strong, weak, and no utilization, respectively. Grey boxes indicate differences between isolates Acjc2 and Acjd4.

### In Vitro Antagonistic Assay against *M. plutonius*

**Figure 2.**
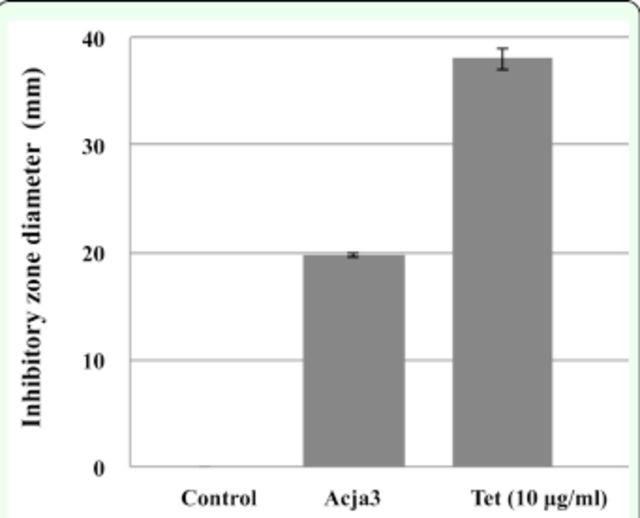
Inhibitory activity of Acja3 isolate obtained from the gut of the Japanese honey bee, *A. c. japonica,* against the *M. plutonius* DAT561 strain. Diameters (mean ± SD) of inhibitory zones were calculated based on three independent experiments. BHI liquid medium and tetracycline (Tet) were used as negative and positive controls, respectively.

To investigate the growth inhibitory activity against the *M. plutonius* DAT561 strain, we subjected all of the gut bacterial isolates to an in vitro inhibitory zone assay. One of the isolates, Acja3, identified as a *Bacillus* strain, showed strong inhibitory activity against the DAT561 strain, and had an average zone diameter of 19.7 mm ( Fig. 2 ). Such inhibitory activity was not observed in any of the other isolates. The positive control (tetracycline) had an average zone diameter of 37.3 mm.

### Infectious Feeding Assay in *A. mellifera* Larvae

**Figure 3.**
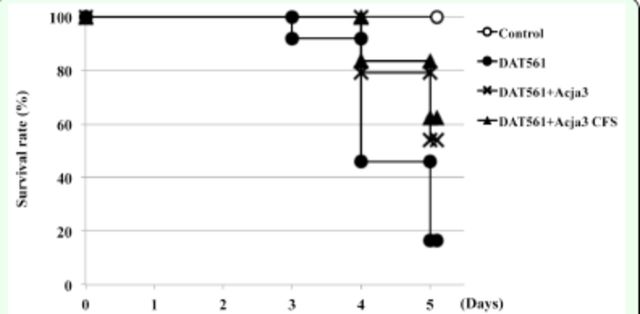
Survival rates of larvae treated with the *M. plutonius* DAT561 strain (solid circles), DAT561 plus Acja3 cell suspension (crosses), DAT561 plus Acja3 cell-free supernatant (CFS) (triangles), or control (open circles). In these in vivo feeding assays, 24 larvae were used in each of the infectious and non-infectious control groups, and 48 larvae were used in each of the Acja3- and Acja3 CFS-treated groups. The average survival rate was used in a log-rank statistical analysis.

*Apis mellifera* is the most commercially important honey bee species. To determine the in vivo antagonistic activity of isolate Acja3 against *M. plutonius,* first-instar larvae of *A. mellifera* were treated with isolate Acja3 or with a cell-free supernatant (CFS) of Acja3 before being administered an artificial diet containing *M. plutonius* DAT561. As shown in Fig. 3 , when larvae were infected with *M. plutonius* DAT561 without any pre-treatments (untreated group), 83.3% of them died within 5 d. However, when larvae were pre-treated with isolate Acja3 or its CFS, 54.2% (Acja3-treated group) and 62.5% (Acja3 CFS-treated group) of larvae were still alive at day 5, and mortality was significantly lower than that in the untreated group (log-rank test, *P* = 0.0181 [Acja3-treated group vs. untreated group]; *P =* 0.0033 [Acja3 CFS-treated group vs. untreated group]). The larvae in the uninfected control group maintained a survival rate of 100% throughout the experiments, and no significant difference was observed in the mortality of the larvae in the Acja3-treated and Acja3 CFS-treated groups (log-rank test, *P =* 1.0000). The results suggested that isolate Acja3 had an antagonistic effect on *M. plutonius* in vivo *.*

## Discussion

In this study, we isolated intestinal bacteria from the Japanese honey bee by using culture-dependent methods to assess their potential antagonistic effects on the pathogenic bacterium *M. plutonius.* Sequence analysis of *16S rRNA* genes revealed that most of the isolated bacteria belonged to the genus *Bacillus,* and in vivo feeding assays showed that one of these *Bacillus* isolates (Acja3) exhibited inhibitory activity against *M. plutonius* in *A. mellifera.*

This abundance of *Bacillus* species corroborated the findings of a previous study on Japanese honey bees ( Yoshiyama and Kimura 2009 ), as well as another culture-dependent study that suggested that bacteria in the genus *Bacillus* are important floral components of the Asian honey bee, *Apis cerana indica* F. (Hymenoptera: Apidae) (Disayathanoowat et al. 2012). The European honey bee, *A. mellifera,* has also been reported to harbor high numbers of *Bacillus* species in the gut ( Gilliam and Morton 1978 ; Gilliam 1979 , 1985; Evans and Armstrong 2005 , 2006; Alippi and Reynaldi 2006 ; Sabaté et al. 2009 ). These results imply that members of the genus *Bacillus* are commonly found in the gut of *Apis* spp.

*Bacillus* species inhabit a variety of environments and play an important role as probiotic organisms in humans and animals because of the many metabolites that they produce ( Hong et al. 2005 , Guo et al. 2006 ). The relationships between honey bees and *Bacillus* bacteria have been extensively examined ( Gilliam 1979 , 1985; Gilliam et al. 1984 ). Most of these studies have reported that the symbiotic *Bacillus* bacteria benefit honey bees by facilitating pollen fermentation, food protection, and disease prevention; for example, *Bacillus subtilis* strains have been shown to inhibit the growth of two other major honey bee pathogens, *P. larvae* and *A. apis* ( Alippi and Reynaldi 2006 , Sabaté et al. 2009 ). In addition, Sabaté et al. (2012) reported that *B. subtilis* strain Mori2 had beneficial effects on colony performance. Our results also support the finding that *Bacillus* species have considerable probiotic potential against a variety of pathogenic bacteria.

Bacteria belonging to the genus *Bacillus* are known to produce a wide variety of antimicrobial and antiviral substances, or metabolites. Previous studies on *B. subtilis* have shown that surfactin, a lipopeptide produced by the bacterium, inhibited honey bee pathogens, including *P. larvae* , *A. apis* and *Nosema ceranae* (Microsporidia: Nosematidae) ( Sabaté et al. 2009 , Porrini et al. 2010 ). In the in vivo feeding assays using Acja3, the cell-free supernatant also conferred larval tolerance to *M. plutonius* , which suggests that the antimicrobial activity of the Acja3 isolate was also due to the presence of antimicrobial compounds or metabolites. The in vivo assays were performed using honey bee larvae that were fed an artificial food that was rich in royal jelly. Interestingly, it has been shown that royal jelly prevents the growth of lactic acid bacteria that had been administered as probiotics to feeding larvae ( Forsgren et al. 2009 ). In this study, feeding assays using royal jelly also had a negative effect on Acja3 survival, preventing bacterial growth in less than 12 h (data not shown). It is considered that the observed antibacterial effect of royal jelly may be due to the presence of antimicrobial peptides or its low pH (4.0); consequently, the probiotic effects of Acja3 inoculation may have been underestimated. Although further studies are required to confirm whether this antagonistic activity is observed in individuals and colonies in the natural environment, it appears that the Acja3 isolate is potentially well suited for use as a biological agent for controlling European foulbrood.

Honey bees can acquire gut microorganisms from the natural environment through foods, such as nectar, pollen, and water. Consequently, the gut flora of honey bees varies according to seasonal or geographical differences in food sources, even among individual honey bees from the same colony ( Gilliam and Valentine 1976 , Mohr and Tebbe 2006 , Moran et al. 2012 ). The profile of gut bacteria may also vary depending on the age and physiological condition of honey bees. Newly emerged workers typically have no or very few gut bacteria ( Martinson et al. 2012 ). In these young workers, the initial uptake of such bacteria likely occurs via contact with collected honey and bee bread and via trophallactic exchange with nestmates. Larvae acquire enteric bacteria when nurse bees feed them by trophallaxis. However, reports of differences in larval gut bacterial profiles imply that the gut bacterial profiles of larvae can be influenced by differences in the microbial communities present in bee bread ( Mohr and Tebbe 2006 , Martinson et al. 2012 ). The observation that certain gut bacteria are maintained in all of the developmental stages of an adult bee, irrespective of differences between species, colonies, and individuals, suggests that distinctive gut bacteria are transferred between generations by eusocial behaviors, such as food exchange between the honey bee populations in a hive ( Martinson et al. 2011, 2012 ; Vasquez et al. 2012 ).

A large-scale study to screen honey bees from different geographic locations and/or at different times would therefore be useful in the search for new biological agents that could potentially be applied to the control of European foulbrood and other honey bee pathogens.
